# Mutation profile and therapeutic implications in Peutz-Jeghers syndrome-associated gastric-type endocervical adenocarcinoma

**DOI:** 10.3389/fonc.2026.1780688

**Published:** 2026-06-18

**Authors:** Tianhui Niu, Xiaofang Liu, Siqi Zhang, Mengnan Yu, Xiaoying Wang, Jinghui Jia

**Affiliations:** 1 Department of Obstetrics, Gynecology and Reproductive Sciences, Air Force Medical University, Air Force Medical Center, People’s Liberation Army, Beijing, China; 2 Department of Ultrasound, Air Force Medical University, Air Force Medical Center, People’s Liberation Army, Beijing, China; 3 Laboratory of clinical medicine, Air Force Medical University, Air Force Medical Center, People’s Liberation Army, Beijing, China

**Keywords:** Claudin 18.2, ERBB3, gastric-type endocervical adenocarcinoma, molecular profiling, Peutz-Jeghers syndrome, precision oncology, targeted therapy

## Abstract

Gastric-type endocervical adenocarcinoma (G-EAC) is a rare, highly aggressive malignancy that is not associated with human papillomavirus (HPV) infection. Its occurrence in patients with Peutz-Jeghers syndrome (PJS) is exceptionally rare but clinically critical. In this preliminary study, we performed comprehensive genomic profiling (CGP) using a targeted next-generation sequencing (NGS) panel and immunohistochemistry (IHC) for key biomarkers on a single, well-characterized patient with stage IB3 PJS-associated G-EAC. The primary objective was to identify actionable genomic alterations and potential therapeutic targets to inform personalized treatment strategies. CGP revealed a pathogenic germline *STK11* p.K84* mutation, alongside somatic *KRAS* p.G12A and *ERBB3* p.R667S mutations. IHC demonstrated strong, diffuse positivity for Claudin18.2 and MUC6, confirming a gastric phenotype, while p16 was negative. The integration of genomic and proteomic data revealed a primary actionable target, Claudin18.2 (a proven target in gastric cancer), alongside potential actionable alterations involving ERBB signaling. This proof-of-concept study may suggest that integrating CGP and IHC in the management of PJS-associated G-EAC is feasible and could potentially inform clinical decision-making. The identification of these targets could suggest a rationale for incorporating molecular profiling into the standard diagnostic workflow for this aggressive malignancy, potentially improving outcomes through precision oncology approaches.

## Introduction

Peutz-Jeghers syndrome (PJS), an autosomal dominant disorder caused by germline *STK11* pathogenic variants, confers a significantly elevated lifetime risk of malignancy (1). While gastrointestinal cancers are most common, female patients face a particularly high risk of gynecologic tract cancers (2). Among these malignancies, gastric-type endocervical adenocarcinoma (G-EAC) is a rare but notoriously aggressive HPV-negative tumor that is not effectively detected by standard cervical cancer screening (3). The convergence of PJS and G-EAC thus presents a formidable diagnostic and therapeutic challenge, often leading to delayed diagnosis and poor outcomes (4).

Recent advances in molecular profiling have begun to unravel the complex genomic landscape of these rare tumors, offering potential avenues for personalized therapy (5). Specifically, larger cohort studies have defined a distinct molecular landscape for G-EAC, frequently characterized by *KRAS* mutations, overexpression of Claudin18.2, associated with its gastric-like differentiation (6), and recurrent genomic alterations involving *ERBB3* (7). Importantly, these features are transitioning from mere biomarkers to clinically actionable targets; both Claudin18.2-targeted therapies and ERBB-directed agents have demonstrated promising clinical applicability in early-phase trials (8, 9). Despite these advances in sporadic G-EAC, the specific genomic landscape of G-EAC arising in the context of PJS remains exceptionally rare and underexplored.

Here, we report a case of stage IB3 G-EAC in a 41-year-old woman with PJS. Through integrated molecular analysis, we identified a pathogenic germline *STK11* mutation alongside somatic *KRAS* and *ERBB3* mutations and strong Claudin18.2 expression. Notably, while the *KRAS* mutation is a recognized driver and Claudin18.2 represents a clear therapeutic target, the identified *ERBB3* p.R667S variant remains of uncertain functional significance. This case highlights the need for a high index of suspicion for G-EAC in PJS patients and suggests that characterizing the specific molecular profile of this scarcely documented population may offer valuable insights for guiding precision management.

## Methods

### Patient selection and clinical data collection

A patient with a known history of PJS was referred to the gynecology department and subsequently diagnosed with stage IB3 G-EAC. Clinical data, including demographics, presentation, imaging findings, surgical pathology, and treatment details, were retrospectively collected from electronic medical records. This study was conducted in accordance with the Declaration of Helsinki and approved by the Institutional Ethics Committee of the Air Force Medical Center (Approval No. KT (Research) 2024-31-PJ01). Written informed consent for publication was obtained from the patient.

### Histopathology and immunohistochemistry

Formalin-fixed, paraffin-embedded (FFPE) tissue sections were stained with Hematoxylin and Eosin (H&E) for routine histopathological examination. Immunohistochemistry (IHC) was performed using an automated staining platform. A panel of antibodies was used, including Claudin18.2, MUC6, MUC5AC, p16, p53, and Ki-67. Appropriate positive and negative controls were used. Detailed information on the primary antibodies, including specific clone names, catalog numbers, commercial manufacturers, and applied dilutions, is provided in **Supplementary Table 1**.

### Genomic profiling and bioinformatics

Genomic DNA (gDNA) was extracted from FFPE tumor tissues, and matched peripheral blood DNA was extracted as a germline control using standard kits (Qiagen). For library preparation, 500 ng of input DNA was used for both the tissue and blood samples. A customized 437-gene panel (GENESEEQPRIME^®^) was used for library preparation and sequencing on an Illumina platform. The average sequencing depths were 1000× for tissue, and 200× for matched germline controls. Raw reads were aligned to the human reference genome (hg19) using BWA-MEM. Somatic variants were identified using VarScan2 by comparing tumor tissue with the matched blood DNA to distinguish germline polymorphisms from somatic mutations. The pathogenic germline *STK11* p.K84* mutation identified by NGS was further validated by Sanger sequencing for clinical confirmation. Mutant allele frequencies (MAF) for somatic mutations were calculated as the ratio of mutant reads to total reads at each locus. Candidate variants were filtered against public databases (dbSNP, 1000 Genomes) and a Chinese CHIP database, with CNVs defined by copy number fold-changes >1.6 (amplification) or <0.6 (deletion), and manually reviewed using IGV.

## Results

### Clinical presentation and imaging

**Figure 1:**
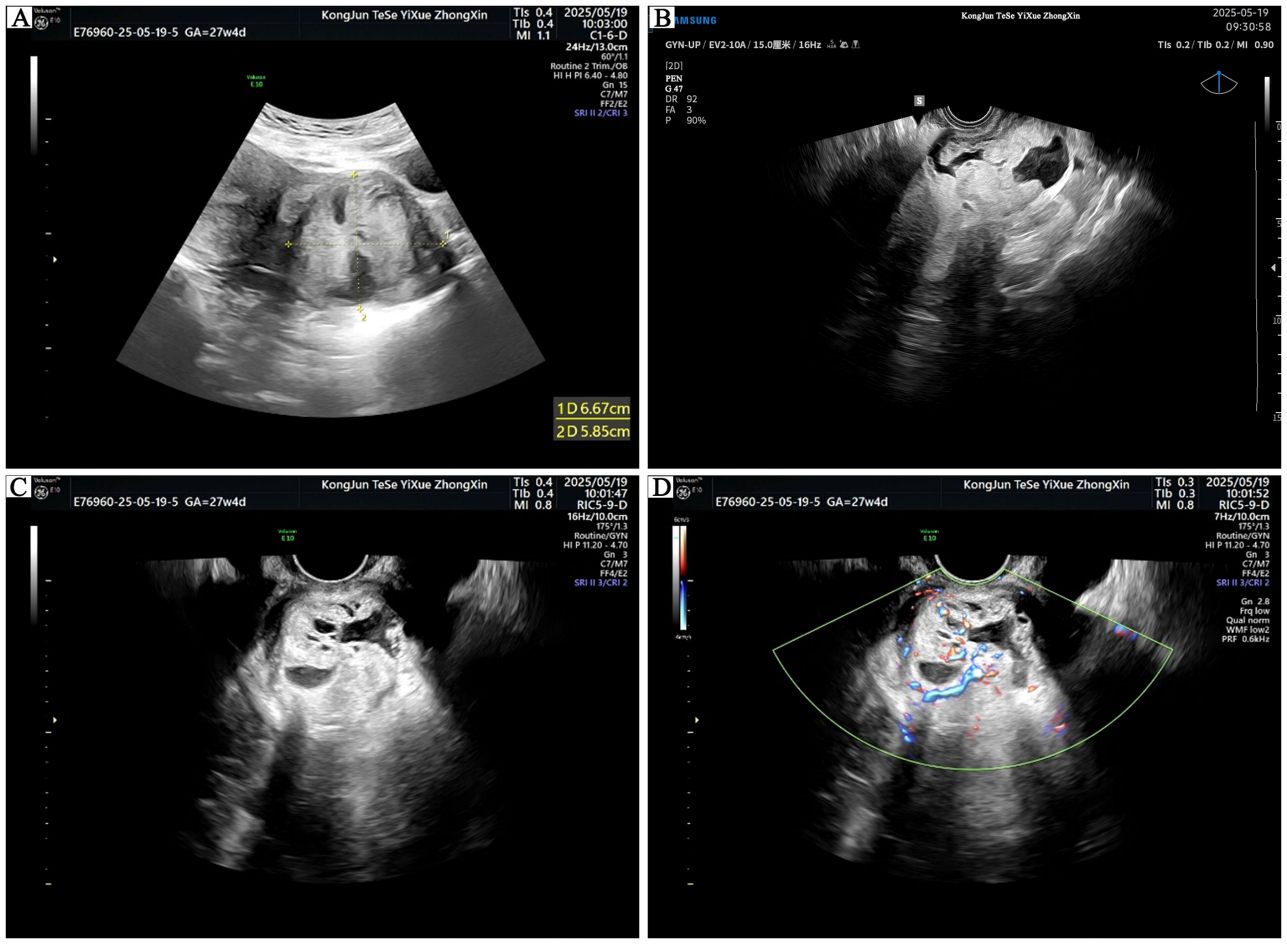
Transvaginal ultrasound findings in a patient with PJS and G-EAC. **(A)** Transverse review reveals a complex cystic-solid cervical lesion measuring 6.9×5.9 cm. **(B)** The lesion demonstrates indistinct boundaries with the lower anterior uterine wall. **(C)** Interventional ultrasound characterizes the lesion as having variably sized cystic areas, irregular margins, and disruption of the cervical serosa. **(D)** Color Doppler imaging demonstrates marked hypervascularity within the solid components of the lesion.

**Figure 2:**
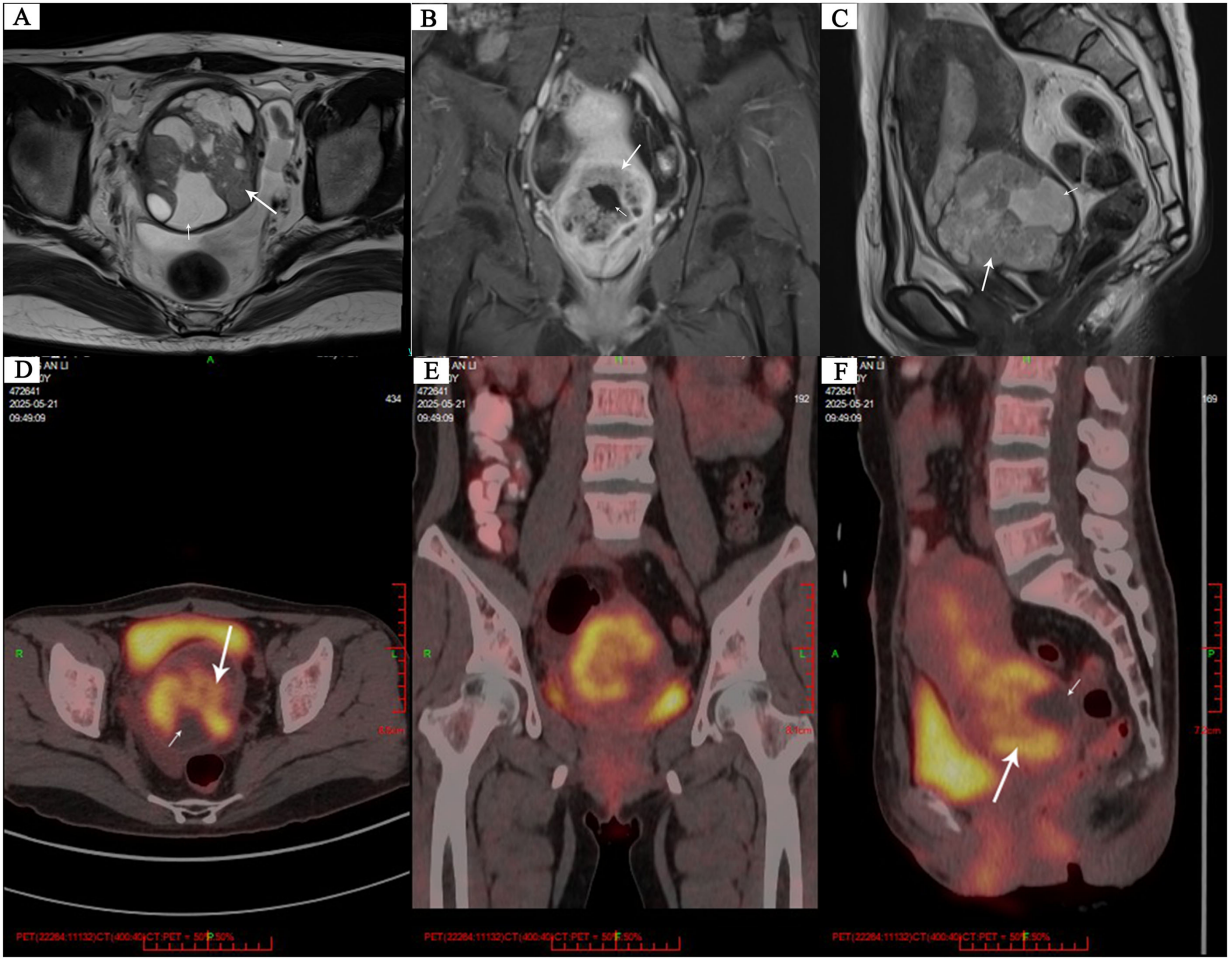
Magnetic resonance imaging (MRI) and 18F-FDG PET/CT findings of the cervical lesion. **(A)** Axial T2-weighted (T2W) image illustrates the cystic-solid nature of the lesion. **(B)** Coronal contrast-enhanced, fat-suppressed T1-weighted (FS-T1W) image demonstrates heterogeneous enhancement of the solid component (long arrow), while the cystic component remains non-enhancing (short arrow). **(C)** Sagittal fat-suppressed T2-weighted (FS-T2W) image provides superior delineation of the lesion’s morphology. **(D-F)** Axial **(D)**, coronal **(E)**, and sagittal **(F)** fused PET/CT images demonstrate intensely increased 18F-FDG uptake (white circle) localized to the solid component of the lesion (long arrows), with a SUVmax of 7.29 and an SUVavg of 4.36. The cystic component (short arrows) shows no significant FDG avidity.

The patient was a 41-year-old female with a known history of PJS who presented with a 3-month history of profuse vaginal discharge. Serum tumor marker analysis revealed an elevated CA19–9 level (264 U/mL). Transvaginal ultrasonography and pelvic MRI revealed a large (7.5 x 5.9 x 6.8 cm) complex cervical mass with irregular borders and local invasion, staged as IB3, along with a suspicious right ovarian mass (**Figures 1**, **2**). An 18F-FDG PET/CT scan demonstrated markedly increased radiotracer uptake (SUVmax 7.29) in the cervical mass and a suspicious right ovarian lesion (**Figure 2**).

### Surgical pathology

**Figure 3:**
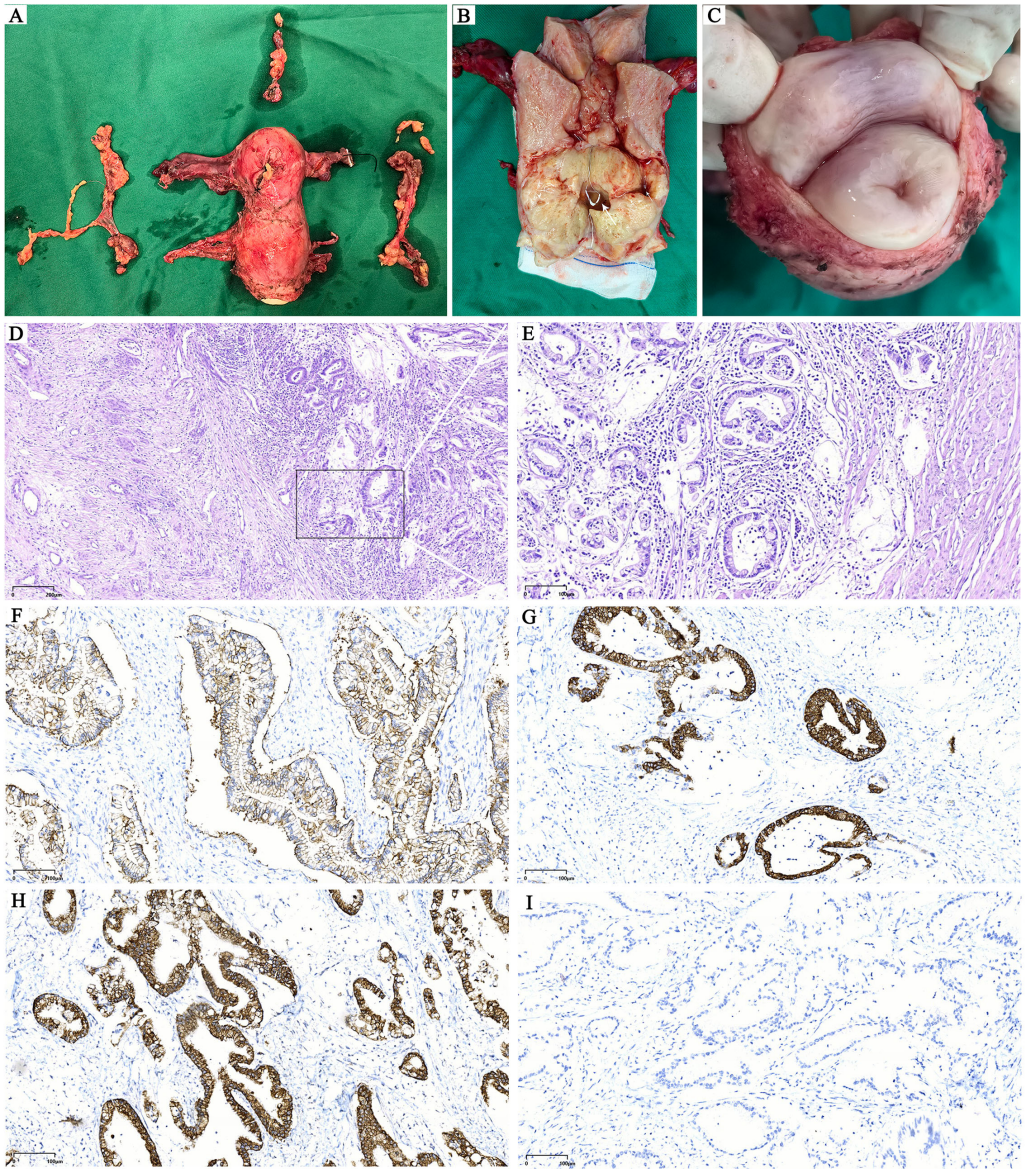
Macroscopic and histopathological findings of the cervical lesion. **(A-C)** Macroscopic examination of the surgical specimen. **(A)** The en bloc resection specimen includes the radical hysterectomy with bilateral salpingo-oophorectomy specimens and pelvic lymphadenectomy. **(B)** The bisected cervix reveals a solid-cystic tumor lesion. A long arrow highlights mucoid discharge from the cervical os, while a short arrow identifies the cystic component. **(C)** Exophytic appearance of the cervical lesion on the ectocervix. **(D, E)** Histopathological examination (H&E stain) reveals tumor cells exhibiting voluminous clear, foamy, or eosinophilic cytoplasm with distinct cell borders. Cytologic atypia is characterized by enlarged, rounded nuclei with distinct nucleoli (Scale bars, 200 µm and 50 µm, respectively). **(F-I)** Immunohistochemistry shows strong positivity for the gastric markers Claudin 18.2 **(F)**, MUC6 **(G)**, and MUC5AC **(H)**. **(I)** Notably, the tumor is negative for p16, consistent with its non-HPV-associated origin (Scale bar, 100 µm).

The patient underwent an abdominal radical hysterectomy with bilateral salpingo-oophorectomy and pelvic lymphadenectomy (**Figure 3**). Final histopathology confirmed the diagnosis of G-EAC, measuring 7.5×5.0×3.5 cm, with deep stromal invasion (>1/2), no lymphovascular space invasion, and involvement of the lower uterine segment. Surgical margins (vaginal cuff and parametria), parametrial tissues, and pelvic lymph nodes were all negative. Notably, the right ovarian mass was identified as a benign lesion. Based on these pathological findings, the final stage was determined as pathological stage IB3 according to the FIGO 2018 classification.

### Genomic and proteomic findings

**Figure 4:**
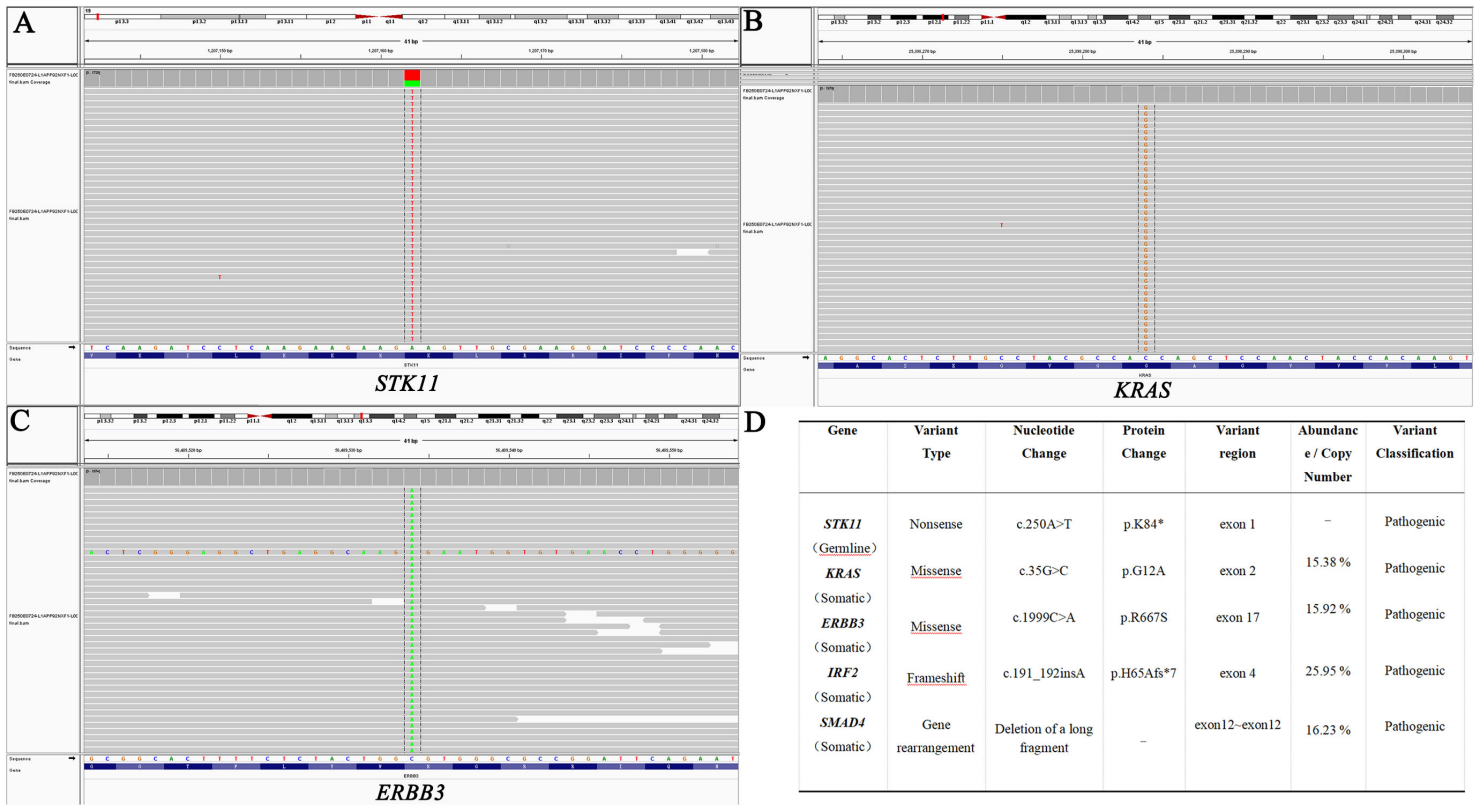
Integrated molecular findings of the G-EAC. Genomic profiling. **(A-C)** Sanger sequencing chromatograms confirm the pathogenic germline *STK11* p.K84* nonsense mutation **(A)**, the somatic KRAS p.G12A **(B)** and *ERBB3* p.R667S missense mutations **(C, D)** Summary table of all identified genetic alterations detected by the NGS panel, including the additional *IRF2* frameshift and *SMAD4* rearrangement.

IHC analysis confirmed the gastric phenotype: strong positivity for Claudin18.2, MUC6, and MUC5AC, with negativity for p16. The Ki-67 proliferation index was approximately 20%, and p53 was wild-type (**Figure 3**). Correspondingly, NGS analysis revealed a pathogenic germline *STK11* p.K84* nonsense mutation, confirming the molecular diagnosis of PJS. Furthermore, two somatic mutations were identified: *KRAS* p.G12A (exon 2, MAF 15.38%) and *ERBB3* p.R667S (exon 17, MAF 15.92%). Further NGS analysis identified two additional pathogenic variants: a somatic frameshift mutation in *IRF2* (p.H65Afs*7, exon 4) and a *SMAD4* rearrangement (a long fragment deletion within exon12) (**Figure 4**). NGS revealed no *ERBB2* amplification, consistent with the negative HER2 status determined by IHC. Moreover, IHC demonstrated a wild-type p53 status, which aligns with the absence of pathogenic variants in the NGS panel (as *TP53* was not included in the sequencing assay) (**Supplementary Table 2**). Finally, NGS analysis did not detect microsatellite instability-high (MSI-H) or pathogenic mutations in mismatch repair (MMR) genes (*MSH2/MSH6*), confirming a microsatellite stable (MSS)/proficient MMR (pMMR) status. PD-L1 expression evaluated by IHC showed low expression with a Combined Positive Score (CPS) of 1%.

### Therapeutic strategy based on molecular profile

Given the IB3 stage with high-risk features (deep stromal invasion), adjuvant chemotherapy was indicated per NCCN guidelines. Due to the lack of G-EAC-specific evidence, a shared decision was made after counseling on the uncertain benefit-risk ratio. The patient received one cycle of carboplatin (500mg, adjusted from the calculated AUC 5–7 dose) and liposomal paclitaxel (180mg), with no recurrence at the 6-month follow-up.

Comprehensive molecular profiling guides future directions. The concurrent MSS/pMMR status, PD-L1 CPS of 1%, and *STK11/KRAS* co-alteration indicate an immunosuppressive microenvironment and likely immunotherapy resistance. Wild-type *TP53* and negative HER2 (confirmed by NGS and IHC) exclude these as targets. Conversely, strong Claudin18.2 positivity and an *ERBB3* p.R667S mutation highlight eligibility for emerging targeted therapies (e.g., zolbetuximab, HER3-directed agents) upon progression, underscoring the need for novel strategies beyond standard chemo-immunotherapy.

## Discussion

This case report provides a detailed molecular profiling of gastric-type endocervical adenocarcinoma (G-EAC) in a patient with Peutz-Jeghers syndrome (PJS), highlighting critical diagnostic and therapeutic challenges. Although the utility of integrating IHC and NGS into the diagnostic workflow, as well as the molecular landscape of G-EAC, has been well-established in larger retrospective studies, the specific genomic profile of G-EAC arising in the context of PJS remains exceptionally rare and scarcely documented. Therefore, rather than proposing a generalized diagnostic paradigm, our case serves as a confirmatory report validating these molecular findings in this specific clinical context. G-EAC is a non-HPV-related adenocarcinoma with an aggressive clinical course and poor prognosis (7). PJS, caused by pathogenic *STK11* variants, is a known cancer predisposition syndrome (10). The identification of a pathogenic germline *STK11* p.K84* mutation in our patient established a definitive molecular link to her PJS, explaining her predisposition to G-EAC within the context of her genetic background and enabling crucial genetic counseling for her family.

Beyond germline predisposition, the somatic mutation profile reveals the tumor’s oncogenic drivers. In addition to the aforementioned *KRAS* and *ERBB3* mutations, NGS identified pathogenic alterations in *IRF2* (frameshift) and *SMAD4* (rearrangement). While their co-occurring in G-EAC is novel, *SMAD4* loss is a known driver of progression and metastasis in gastrointestinal malignancies (11). Notably, the *IRF2* mutation introduces a biological paradox: although tumor-intrinsic *IRF2* loss promotes immune evasion, recent evidence demonstrates that its inactivation prevents CD8+ T cell exhaustion and enhances anti-tumor immunity (12). This duality highlights the complex interplay between tumor genomics and the immune microenvironment. Genomic-IHC correlation was further validated by the absence of *TP53* mutations and *ERBB2* amplification, which aligned with the wild-type p53 and negative HER2 IHC findings. Finally, the concurrent *KRAS* p.G12A mutation represents a classic oncogenic driver in PJS-associated G-EAC (13). While this variant potentially confers sensitivity to MEK inhibitors, KRAS-driven immune evasion strongly suggests primary resistance to immunotherapy (14, 15). The therapeutic implications of these genomic profiles are discussed below.

More intriguingly, the identification of a somatic *ERBB3* p.R667S missense mutation adds another layer of complexity. *ERBB3* (HER3), a member of the HER family, possesses an impaired kinase domain and signals through heterodimerization, particularly with HER2 (16). While the oncogenic potential of *ERBB3* mutations is an area of active investigation, the specific biological role of the p.R667S variant remains unclear. A recent comprehensive functional study of *ERBB3* pseudokinase domain mutations did not identify p.R667S as a pathogenic or activating variant, classifying its functional significance as uncertain (17). Therefore, while this specific mutation could represent a potential therapeutic target—rendering the tumor susceptible to HER2/HER3-directed agents, such as monoclonal antibodies or antibody-drug conjugates (ADCs)—this remains hypothetical. Our study confirms the recurrent molecular alterations in G-EAC, including *STK11*, *KRAS*, and *ERBB3*, and specially documents the rare co-occurrence of these alterations in a PJS patient. This combined *STK11/KRAS/ERBB3* mutation profile has profound implications for future therapeutic strategies.

Diagnostically, this case was challenging. While imaging defined the extent of the disease, the non-specific presentation of vaginal discharge in a high-risk PJS patient necessitates a high index of suspicion. Although elevated CA199 provided a clue, it is important to note that this marker lacks specificity and is not uniformly elevated in all G-EAC cases. Therefore, definitive diagnosis relied on a comprehensive IHC panel. Positivity for gastric markers (Claudin18.2, MUC6, and MUC5AC) and negative for p16 were crucial in this specific case for distinguishing G-EAC from its mimics and confirming its non-HPV etiology (18, 19). Notably, the expression of Claudin18.2 in G-EAC is pathologically consistent with its gastric epithelium-like differentiation, a phenomenon recently highlighted across various tumor types (6). This underscores our recommendation that for known PJS patients, any gynecologic symptom should prompt a low threshold for definitive tissue diagnosis, potentially including a cervical conization or direct biopsy, moving beyond standard HPV-based screening paradigms (20).

Therapeutically, G-EAC demands a rigorous, multidisciplinary approach. For advanced or recurrent G-EAC, guideline-recommended treatments primarily rely on platinum-based chemotherapy, often combined with bevacizumab or pembrolizumab; however, the tumor is highly chemoresistant, and immunotherapy efficacy remains limited in this non-HPV-driven subtype. Given its propensity for ovarian metastasis, pelvic spread, and recurrence, meticulous imaging and radical surgery with negative margins are paramount (21). Despite inherent chemoresistance, adjuvant platinum-based chemotherapy was administered given the pathological stage IB3 and aggressive tumor biology. Importantly, due to the lack of G-EAC-specific evidence, this decision was made through shared decision-making, ensuring transparent communication of uncertainties to obtain truly informed consent. The molecular findings, however, open a spectrum of future options. Strong Claudin18.2 positivity presents a highly promising therapeutic target (22), with Claudin18.2- directed agents currently being explored in gastric cancer and pan-tumor basket trials for Claudin18.2-positive solid tumors (8). Similarly, HER3-directed antibody-drug conjugates (ADCs) are under investigation in biomarker-driven trials for solid tumors harboring *ERBB3* alterations (9).

Importantly, the identified actionable targets (Claudin18.2 and *ERBB3*) did not guide clinical decisions, as corresponding targeted agents lack approved indications in cervical adenocarcinoma and the oncogenic role of *ERBB3* p.R667S remains uncertain. Consequently, standard NCCN-guided adjuvant chemotherapy was prioritized. This highlights a persistent translational gap: while molecular profiling illuminates future therapeutic avenues, its direct applicability in G-EAC requires validation in larger cohorts. Furthermore, comprehensive biomarker profiling definitively excluded alternative targeted or immunotherapeutic options. Dual genomic and IHC analyses confirmed an MSS phenotype, wild-type *TP53*, and the absence of *ERBB2* amplication, precluding HER2-targeted therapies. Crucially, although the PD-L1 CPS of 1% technically meets the threshold for immune checkpoint inhibitors eligibility in cervical cancer, this is offset by concurrent MSS status and *STK11/KRAS* co-alteration—genomic hallmarks that drive a profoundly immunosuppressive tumor microenvironment and primary immunotherapy resistance (23). This confluence of an immunologically “cold” phenotype and limited targeted options underscores the adverse prognostic implications and amplifies the rationale for exploring novel Claudin18.2- or *ERBB3*-directed strategies beyond conventional chemo-immunotherapy.

Interestingly, the co-expression of Claudin18.2 and the *ERBB3* p.R667S mutation suggests a potential molecular synergy, which may further enhance the tumor’s susceptibility to dual-targeted approaches, particularly in light of the proven efficacy of Claudin18.2-directed therapies in gastric cancer (24). The *KRAS* mutation, while challenging, may guide future clinical trial eligibility, and the identified *ERBB3* mutation warrants further exploration as a potential target (25), provided its functional significance can be clarified.

In conclusion, this confirmatory case report underscores three important clinical considerations for this rare subset of patients: (1) heightened oncological vigilance in PJS patients, employing advanced imaging when needed (26); (2) diagnostic precision via deep tissue biopsy and comprehensive IHC as previously established (27); and (3) multidisciplinary management integrating surgery, oncology, and genetics (28). The confirmation of a multi-faceted molecular profile—germline *STK11* loss combined with somatic *KRAS* and *ERBB3* mutations, alongside Claudin18.2 positivity—transforms this rare clinical scenario into a compelling opportunity for precision medicine. It may suggest that characterizing the specific molecular profile of PJS-associated G-EAC could offer valuable insights for managing this challenging disease, guiding both current management and paving the way for future, personalized therapeutic strategies (29). Admittedly, as a confirmatory report based on a single case, the clinical efficacy of targeting these identified alterations remains to be validated in larger patient cohorts. Nevertheless, the distinct co-occurrence of *STK11* loss, *KRAS* activation, *ERBB3* mutation, and Claudin18.2 expression provides a valuable molecular blueprint for future biological research and the design of basket trials targeting this aggressive subset of cervical cancer.

## Data Availability

The original contributions presented in the study are included in the article/**Supplementary Material**. Further inquiries can be directed to the corresponding author.
