# Supplementary material for: Mutation profile and therapeutic implications in Peutz-Jeghers syndrome-associated gastric-type endocervical adenocarcinoma
Source: Front Oncol. 2026 Jun 18;16:1780688. doi: 10.3389/fonc.2026.1780688 (PMC13323026; doi:10.3389/fonc.2026.1780688)
Supplement: Supplementary file 1 [file Table1.docx]

1. **Table S1. Details of antibodies used for immunohistochemical (IHC) staining.**This table lists the primary antibodies employed in the study, including the targeted markers, specific clone names, catalog numbers, commercial manufacturers, and the applied dilutions. All antibodies utilized in this study were prediluted ready-to-use formulations.

| **Antibody** | **Clone** | **Cat. No.** | **Manufacturer** | **Dilution** |
| --- | --- | --- | --- | --- |
| Claudin18.2 | ZR451 | GC-03048M | Golden Bridge Ultra | Ready-to-use |
| MUC6 | OTIR4C11 | ZA-0697 | ZSGB-BIO | Ready-to-use |
| MUC5AC | EP362 | ZA-0664 | ZSGB-BIO | Ready-to-use |
| p16 | JC2 | GP-1660R | Golden Bridge Ultra | Ready-to-use |
| Ki-67 | umab107 | ZM-0166 | ZSGB-BIO | Ready-to-use |
| p53 | DO-7 | GP-1648 | Golden Bridge Ultra | Ready-to-use |
| HER2 | 4B5 | 1. 790-4493 | Roche | Ready-to-use |

Scoring systems and cut-offs for key immunohistochemical markers:

**Claudin18.2**: Membranous staining in ≥1% of tumor cells was considered positive, consistent with eligibility criteria for Claudin18.2-targeted clinical trials.

**p16**: Block-positive nuclear and cytoplasmic staining was interpreted as positive (surrogate for HPV integration); complete absence or focal/patchy staining was interpreted as negative.

**p53**: Wild-type pattern was defined as patchy, variable nuclear staining. Mutant patterns included diffuse strong overexpression or complete null expression.

**Ki-67**: The proliferation index was determined by counting positive tumor cell nuclei in the highest labeling areas (hot spots) and reported as a percentage.

**HER2**: Scoring was based on the ASCO/CAP guidelines for gastric/gastroesophageal adenocarcinoma. Negative: 0 or 1+; Equivocal: 2+; Positive: 3+.

1. **Table S2. Comprehensive molecular profiling and immunohistochemical correlation of key biomarkers.**This table presents the dual validation of ERBB2 (HER2) and TP53 status by integrating both genomic and proteomic data within the same row. For each biomarker, the columns sequentially detail the Next-Generation Sequencing (NGS) findings—including variant type, nucleotide and protein changes, variant region, copy number/abundance, and variant classification—alongside the corresponding phenotypic immunohistochemical (IHC) results. This integrated layout demonstrates a precise concordance: the absence of ERBB2 amplification by NGS directly corresponds with negative HER2 protein expression by IHC, and the wild-type TP53 genomic status aligns with the wild-type p53 immunophenotype. The detection methods and clinical relevance of each biomarker are also indicated.

| **Gene** | **Variant**  **Type** | **Nucleotide**  **Change** | **Protein**  **Change** | **Variant**  **region** | **Abundance / Copy Number** | **Variant Classification** | **Detection Method** | **Clinical Relevance** |
| --- | --- | --- | --- | --- | --- | --- | --- | --- |
| ***ERBB2***  （HER2）  TP53 | CNV  Wild-type | —  — | —  — | —  — | No amplification  Wild-type | Benign  (No amplification)  Wild-type (No pathogenic variants) | IHC  IHC | HER2 status (therapeutic target)  p53 status (prognostic marker) |
